# Predicting SARS-CoV-2 infection among hemodialysis patients using deep neural network methods

**DOI:** 10.1038/s41598-024-74967-4

**Published:** 2024-10-09

**Authors:** Lihao Xiao, Hanjie Zhang, Juntao Duan, Xiaoran Ma, Len A. Usvyat, Peter Kotanko, Yuedong Wang

**Affiliations:** 1 Department of Statistics and Applied Probability, University of California, Santa Barbara, CA USA; 2 Renal Research Institute, New York, USA; 3 Fresenius Medical Care, Waltham, MA USA; 4 Icahn School of Medicine at Mount Sinai, New York, USA

**Keywords:** Medical research, Nephrology

## Abstract

COVID-19 has a higher rate of morbidity and mortality among dialysis patients than the general population. Identifying infected patients early with the support of predictive models helps dialysis centers implement concerted procedures (e.g., temperature screenings, universal masking, isolation treatments) to control the spread of SARS-CoV-2 and mitigate outbreaks. We collect data from multiple sources, including demographics, clinical, treatment, laboratory, vaccination, socioeconomic status, and COVID-19 surveillance. Previous early prediction models, such as logistic regression, SVM, and XGBoost, require sophisticated feature engineering and need improved prediction performance. We create deep learning models, including Recurrent Neural Networks (RNN) and Convolutional Neural Networks (CNN), to predict SARS-CoV-2 infections during incubation. Our study shows deep learning models with minimal feature engineering can identify those infected patients more accurately than previously built models. Our Long Short-Term Memory (LSTM) model consistently performed well, with an AUC exceeding 0.80, peaking at 0.91 in August 2021. The CNN model also demonstrated strong results with an AUC above 0.75. Both models outperformed previous best XGBoost models by over 0.10 in AUC. Prediction accuracy declined as the pandemic evolved, dropping to approximately 0.75 between September 2021 and January 2022. Maintaining a 20% false positive rate, our LSTM and CNN models identified 66% and 64% of positive cases among patients, significantly outperforming XGBoost models at 42%. We also identify key features for dialysis patients by calculating the gradient of the output with respect to the input features. By closely monitoring these factors, dialysis patients can receive earlier diagnoses and care, leading to less severe outcomes. Our research highlights the effectiveness of deep neural networks in analyzing longitudinal data, especially in predicting COVID-19 infections during the crucial incubation period. These deep network approaches surpass traditional methods relying on aggregated variable means, significantly improving the accurate identification of SARS-CoV-2 infections.

## Introduction

Over the past few decades, Time Series Classification (TSC) has emerged as a prominent subject of research interest. TSC entails a supervised learning task wherein sequences of temporal observations are used as inputs, generating discrete class labels that characterize the input time series. These time series can encompass various data types, ranging from hourly temperature records to stock market data. Time series analysis has garnered substantial attention and exploration across diverse domains, including healthcare, finance, industrial engineering, and computer science. Within the medical realm, patient medical histories are frequently recorded as time series and stored in their Electronic Health Records (EHRs). By analyzing these EHR time series data, it is possible to identify the presence of specific diseases^1^.

The surge in the volume and intricacy of time series data has prompted the adoption of deep learning techniques for a diverse range of classification tasks. Among these techniques, Deep Convolutional Neural Networks (CNNs) and Recursive Neural Networks (RNNs) have garnered significant attention for their ability to extract complex features and temporal correlations from raw time series data. CNNs apply convolutional filters to the input data, effectively capturing local dependencies and hierarchical patterns, which are crucial for distinguishing subtle variations in time series data. As a gated RNN, Long Short-Term Memory network (LSTM)^2^ incorporates a memory cell that can maintain its state over long sequences, along with gates that regulate the flow of information, thus adeptly capturing long-term dependencies both long-term and short-term dependencies across various real-world scenarios. These networks have been successfully employed in numerous applications such as activity recognition and action recognition^3^, time series classification^4,5^, machine translation^6^, and stock price prediction^7^. Their ability to unravel intricate patterns and relationships within time series data makes them state-of-the-art methods in contemporary classification endeavors.

As the COVID-19 pandemic unfolded, deep learning models were frequently employed by the medical research community for various COVID-related analyses. For example, CNN and LSTM have been used to analyze chest radiographs (CXR) and computed tomography (CT) images to detect COVID-19 cases^8–11^. Leveraging the sequential nature of electronic health records (EHR), Bert-based models have been used for early detection^12^, and RNNs are trained to predict COVID-19 disease progression and patient outcome^13,14^.

COVID-19 has created more devastation to dialysis patients than the general population during the pandemic. Predicting COVID-19 outbreaks and monitoring the virus’s impact on dialysis clinics is important for several reasons. In general, dialysis patients have compromised immune systems, which increases their susceptibility to severe complications if they contract COVID-19^15^. The ability to predict outbreaks enables clinics to implement preventive measures to safeguard these vulnerable patients proactively. Furthermore, accurate prediction is crucial in helping clinics allocate resources efficiently. For example, when an outbreak is anticipated, clinics can take proactive steps to ensure adequate testing kits and medical staff to manage the potential increase in cases. This preparedness is essential for timely and effective patient care while minimizing the risk of further spread within the clinic. Previous COVID-19 early prediction models for dialysis population are limited to gradient-boosted decision trees or other traditional machine learning methods^15–17^. One possible reason for that is neural networks typically demand substantial datasets for effective training. Fortunately, a sizable dataset is readily accessible from the clinic’s patient records, encompassing COVID-19 test outcomes, patient demographics, and clinic-specific parameters. In addition, county-level infection data are publicly available. Our goal is to develop DNNs that incorporate multi-modal data to improve prediction accuracy.

## Results

We established a cohort consisting of 41,515 COVID-19-positive patients and 114,119 unaffected patients. The demographic and categorical features of hemodialysis patients with and without SARS-CoV-2 infection are summarized in Table 1. Similarly, Table 2 presents 28 numerical treatment and county-level input features, and Table 3 presents 25 numerical lab input features.

**Table 1:** Demographics and categorical features of hemodialysis patients with and without SARS-CoV-2 infection.

Variable [abbreviation]	Unaffected patients	COVID-19 positive patients
Number of patients on HD	114,119	41,515
Age (year)	63.67	62.84
Dialysis vintage (year)	3.54	4.16
Fully vaccinated, n (%) [get_vac]	13,162 (11.5)	4647 (11)
Number of days since the last vaccination (day) [last_vac_to_pcr]	21	31
Male, n (%)	67,717 (59)	22,857 (55)
Hispanic or Latino, n (%)	15,748 (14)	7625 (19)
Nursing Home, n (%)	6025 (6)	5290 (15)
Asthma, n (%)	4183 (4)	1634 (4)
Cancer, n (%)	5640 (5)	1700 (4)
COPD, n (%)	12,343 (11)	4686 (11)
Diabetes, n (%)	48,928 (45)	19,256 (49)
Hypertension, n (%)	82,510 (72)	30,833 (74)
Immunosupp, n (%)	164 (0.1)	58 (0.1)
Mental illness, n (%)	16,691 (15)	6674 (16)
Pregnancy, n (%)	63 (0.05)	39 (0.09)
Race, n (%)		
American Indian or Alaska Native	897 (1)	597 (1)
Asian	4565 (4)	988 (2)
Black or African American	39,942 (35)	14,115 (34)
Native Hawaiian or Other Pacific Islander	1385 (1)	491 (1)
White	67,330 (59)	25,324 (61)
Education,n (%)		
8 or less years of school	7201 (7)	4289 (10)
Current student	78 (0)	31 (0)
GED	2951 (3)	1142 (3)
Graduated from 2 or 4 year college	17,563 (15)	4871 (12)
Graduated high school	44,257 (38)	16,125 (39)
Graduate school	4960 (4)	1135 (3)
More than 8 years but less than 12	14,252 (12)	6390 (15)
Some college	18,677 (16)	6156 (15)
Vocational/Technical school	4074 (4)	1364 (3)
Other	106 (0)	12 (0)
Insurance type, n (%)		
Type 1 [ins_type1]	8132 (7.2)	2915 (7)
Type 2 [ins_type2]	104,334 (91.4)	38,109 (91.8)
Type 3 [ins_type3]	759 (0.7)	350 (0.8)
Type Unknown	894 (0.7)	142 (0.4)

**Table 2:** Numerical treatment and county-level Input features of hemodialysis patient with and without SARS-CoV-2 infection.

Variable [abbreviation]	Unaffected patients	COVID-19 positive patients
	Mean ± SD	Mean ± SD
County-level local information		
Population number [pop_num]	1.09 × 10^6^ ± 1.78 × 10^6^	1.14 × 10^6^ ± 1.87 × 10^6^
Population density [pop_density]	1241 ± 2630	1303 ± 2987
PCT-Poverty	13.63 ± 5.14	14 ± 5.17
Daily infected COVID cases [covid_new_cases]	503 ± 2468	1065 ± 3987
Daily COVID decease [covid_death]	1934 ± 4338	1590 ± 3838
Treatment information		
Target weight (kg)	3.29 ± 19.73	3.33 ± 21.34
Average QB (mL/min)	405.57 ± 56.82	408.60 ± 56.62
Average QD (mL/min)	716.54 ± 100.30	714.96 ± 101.28
Pre-HD Weight (kg)	85.55 ± 24.09	86.82 ± 24.87
Post-HD Weight (kg)	83.33 ± 23.61	84.66 ± 24.39
IDWG (kg)	2.24 ± 1.41	2.20 ± 1.5
Pre-HD sitting SBP (mm Hg)	148.24 ± 25.82	147.98 ± 26.63
Post-HD sitting SBP (mm Hg)	139.07 ± 23.9	141.23 ± 24.74
Pre-HD sitting DBP (mm Hg)	77.27 ± 15.6	76.70 ± 15.95
Post-HD sitting DBP (mm Hg)	73.65 ± 14.1	74.03 ± 14.47
Pre-HD body temperature ($^{\circ }{\textrm{F}}$) [pre_temp]	97.38 ± 0.76	97.46 ± 0.84
Post-HD body temperature ($^{\circ }{\textrm{F}}$) [post_temp]	97.45 ± 0.69	97.53 ± 0.79
Pre-HD pulse (BPM)	78.88 ± 13.14	79.59 ± 13.41
Post-HD pulse (BPM)	76.03 ± 12.616	77.25 ± 13.1
Pre-HD respiratory (mm Hg) [pre_resp]	17.62 ± 1.49	17.62 ± 1.49
Post-HD respiratory (mm Hg) [post_resp]	17.54 ± 1.47	17.55 ± 1.47
Min-HD sitting SBP (mm Hg)	117.87 ± 23	119.02 ± 24.2
Max-HD sitting SBP (mm Hg)	155.43 ± 24.96	157.89 ± 25.75
Min-HD sitting DBP (mm Hg)	62.74 ± 13	62.87 ± 13.44
Max-HD sitting DBP (mm Hg)	84.46 ± 16.72	85.31 ± 17.31
Min-HD pulse (BPM)	65.83 ± 11.48	66.81 ± 11.90
Max-HD pulse (BPM)	80.36 ± 13.96	81.85 ± 14.73
KTV	1.64 ± 0.35	1.64 ± 0.36

**Table 3:** Numerical Lab Input features of hemodialysis patient with and without SARS-CoV-2 infection.

Variable [abbreviation]	Unaffected patients	COVID-19 positive patients
	Mean ± SD	Mean ± SD
Lab information		
Albumin (g/dl)	3.83 ± 0.41	3.72 ± 0.45
Creatinine (mg/dL)	8.21 ± 3.09	8.20 ± 3.09
Chloride (mEq/L)	99.19 ± 4.12	98.98 ± 4.18
BUN (mg/dL)	56.39 ± 18.57	55.41 ± 18.90
Sodium (mEq/L)	137.71 ± 3.27	137.53 ± 3.40
TSAT (%)	32.63 ± 14.03	31.48 ± 14.48
Potassium (mEq/L)	4.74 ± 0.68	4.76 ± 0.69
Ferritin (ng/mL)	837.09 ± 409.3	886 ± 411
PTH (pg/mL)	441.86 ± 322	411 ± 330
nPCR (g/kg/day)	0.98 ± 0.35	0.96 ± 0.46
Calcium (mg/dl)	8.95 ± 0.69	8.84 ± 0.71
Platelets (mg/dl)	197.71 ± 74.9	196.29 ± 79.83
Phosphorus (mg/dl)	5.47 ± 1.68	5.41 ± 1.76
Hgb (g/dl)	10.72 ± 1.28	10.63 ± 1.29
WBC count ($10^{10}$ /L)	6.92 ± 3.19	6.81 ± 3.13
Neutrophils (mg/dl)	66.19 ± 9.86	67.34 ± 10.19
Lymphocytes (%)	20.04 ± 8.21	19 ± 8.4
NLR	4.37 ± 3.49	4.79 ± 3.93
Monocytes (%)	6.39 ± 1.96	6.58 ± 2.19
Eosinophils (%)	4.30 ± 2.64	3.99 ± 2.63
Basophils (%)	0.80 ± 0.66	0.78 ± 0.65
URR (%)	74.60 ± 6.7	74.71 ± 6.5
Hepatitis B surface antibody (IU/L) [hepb_antihbs]	235.23 ± 347.55	219.16 ± 337.72
Pre-oxygen (%) [pre_O2]	96.17 ± 4.69	96 ± 4.44
Post-oxygen (%) [post_O2]	95.54 ± 6.87	95.46 ± 6.63

**Fig. 1:**
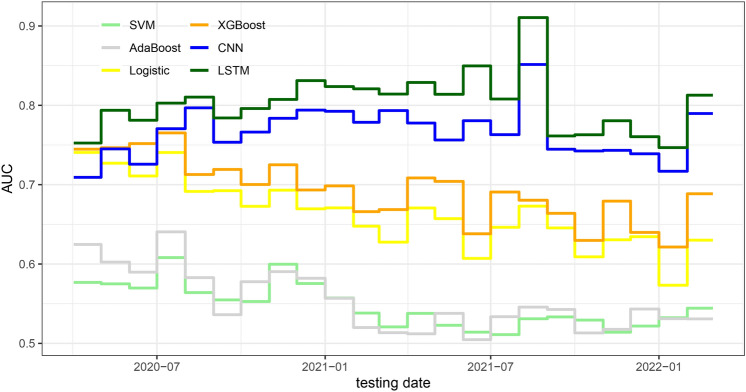
Monthly AUCs from LSTM, CNN, XGBoost, Logistic Regression, AdaBoost, and SVM models based on cumulative data.

Figure 1 indicates that the LSTM model based on cumulative data has the best performance with an AUC above 0.75 throughout the whole time period. Its performance kept increasing roughly until August 2021 and dropped afterward, perhaps due to degraded data quality since the CDC switched COVID-19 surveillance from daily to weekly reports in October 2021. The CNN model based on cumulative data exhibits a similar trend over time, with AUC values consistently below 0.80. Both RNN and CNN models outperform the XGBoost model used in Monaghan et al.^15^ and Duan et al.^17^. The improvement is substantial, with the LSTM model achieving AUC values consistently 0.1 to 0.15 higher than the XGBoost model. The largest improvement was observed in August 2021, where the difference in AUCs of the LSTM and XGBoost models was 0.22. These results underscore the effectiveness of the proposed approach in enhancing COVID-19 detection performance. LSTM also outperforms a set of traditional machine learning models, such as Logistic Regression, SVM, and AdaBoost, by a large margin, reflecting its advantage in effective pattern recognition from time series data.

**Fig. 2:**
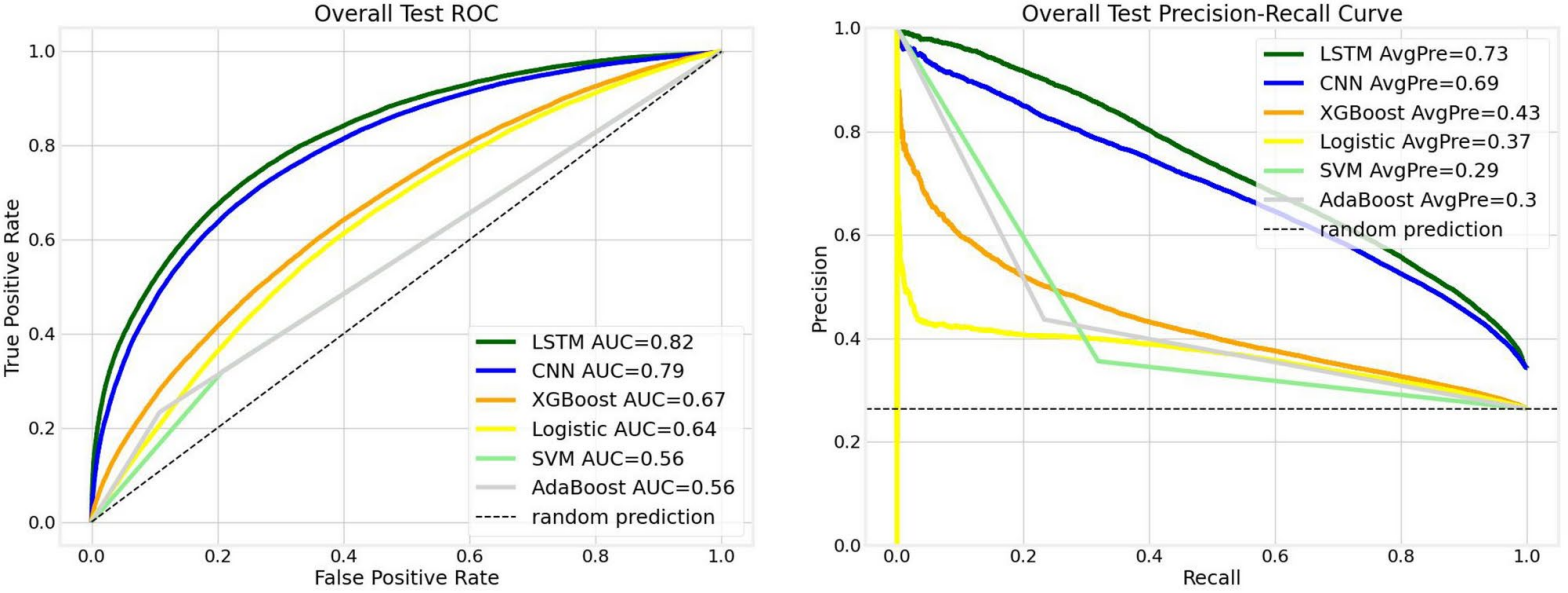
Overall testing performance is calculated with aggregated monthly predictions. We compare receiver operating characteristic curves (AUCs) (on the left) and precision-recall curves (PRCs) (on the right) for LSTM, CNN, and XGBoost models based on cumulative data. AvgPre is the average precision.

Figure 2 shows the overall prediction performance aggregating predictions across all individual months. The overall precision is calculated as the ratio of correctly predicted positive COVID-19 cases to the total number of positive COVID-19 cases observed throughout the testing period. The LSTM model slightly outperforms the CNN, achieving AUC and precision scores of 0.82 and 0.73, respectively, compared to 0.79 and 0.69 for the CNN. Both the LSTM and CNN models demonstrate a significant performance enhancement over XGBoost, Logistic Regression, AdaBoost, and SVM.

### Feature importance

Feature importance in deep neural networks can be effectively analyzed using gradient-based methods^18–20^. This approach involves computing the average gradient of the testing loss function with respect to each input feature across multiple objects/patients. This technique has proven to provide a clear understanding of feature relevance, thereby enhancing the interpretability of RNN models by highlighting the features that most significantly impact the model’s performance.

**Fig. 3:**
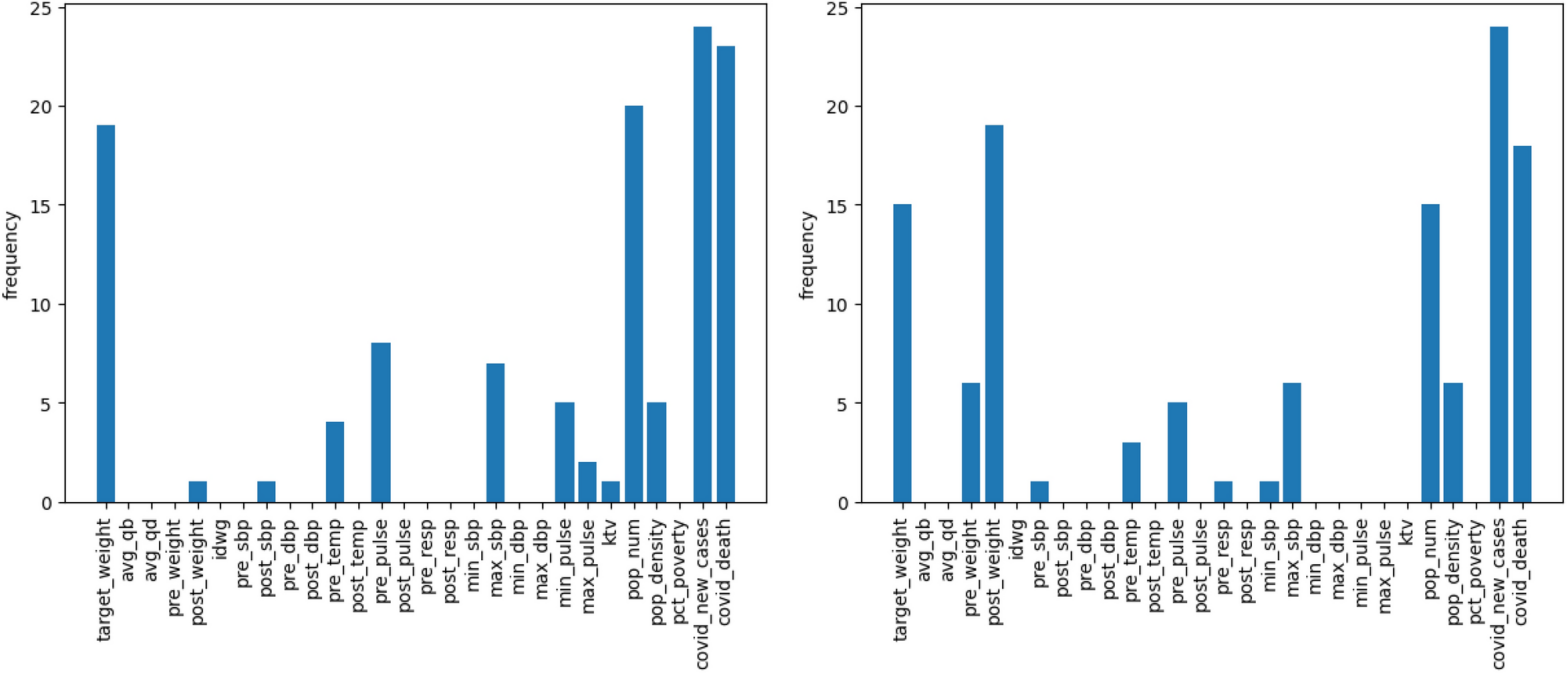
The number of months over the 25-month period for predictors are among the top five important treatment and local features for LSTM (left) and CNN (right).

**Fig. 4:**
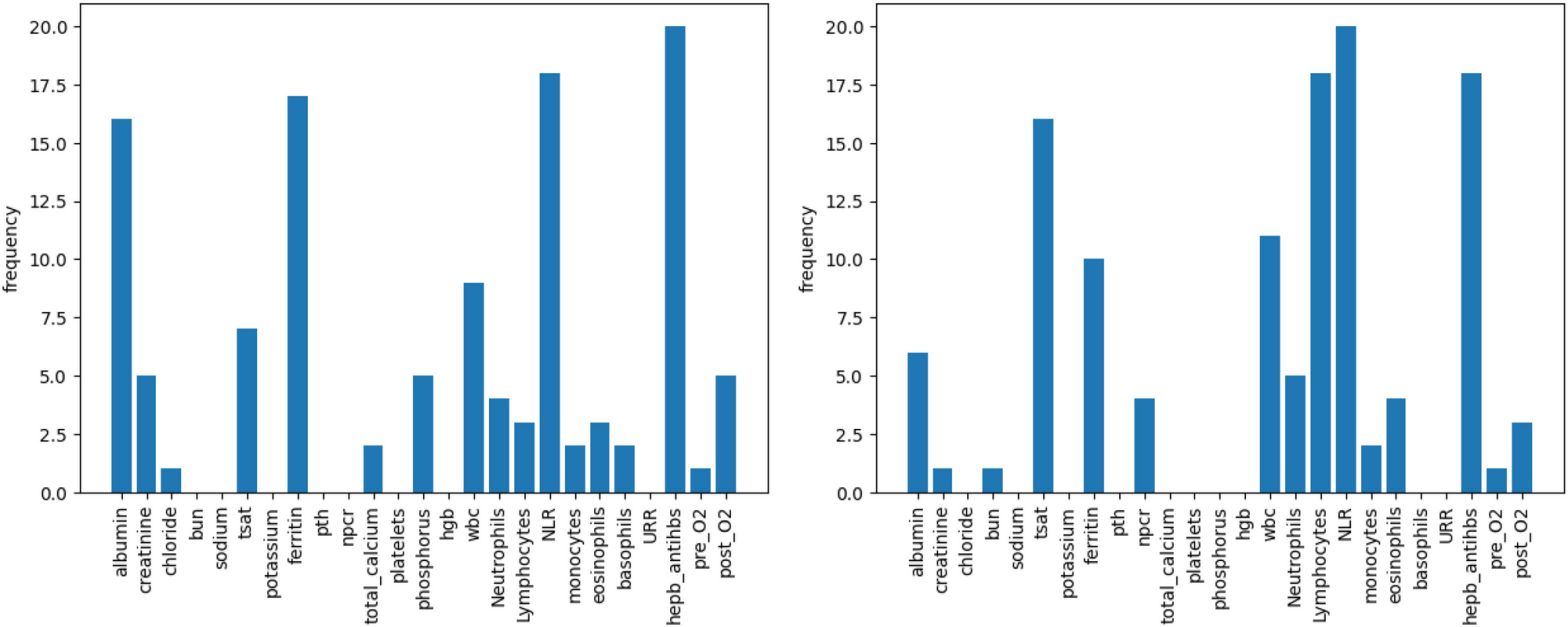
The number of months over the 25-month period for predictors are among the top five important lab variables in LSTM(left) and CNN(right).

**Fig. 5:**
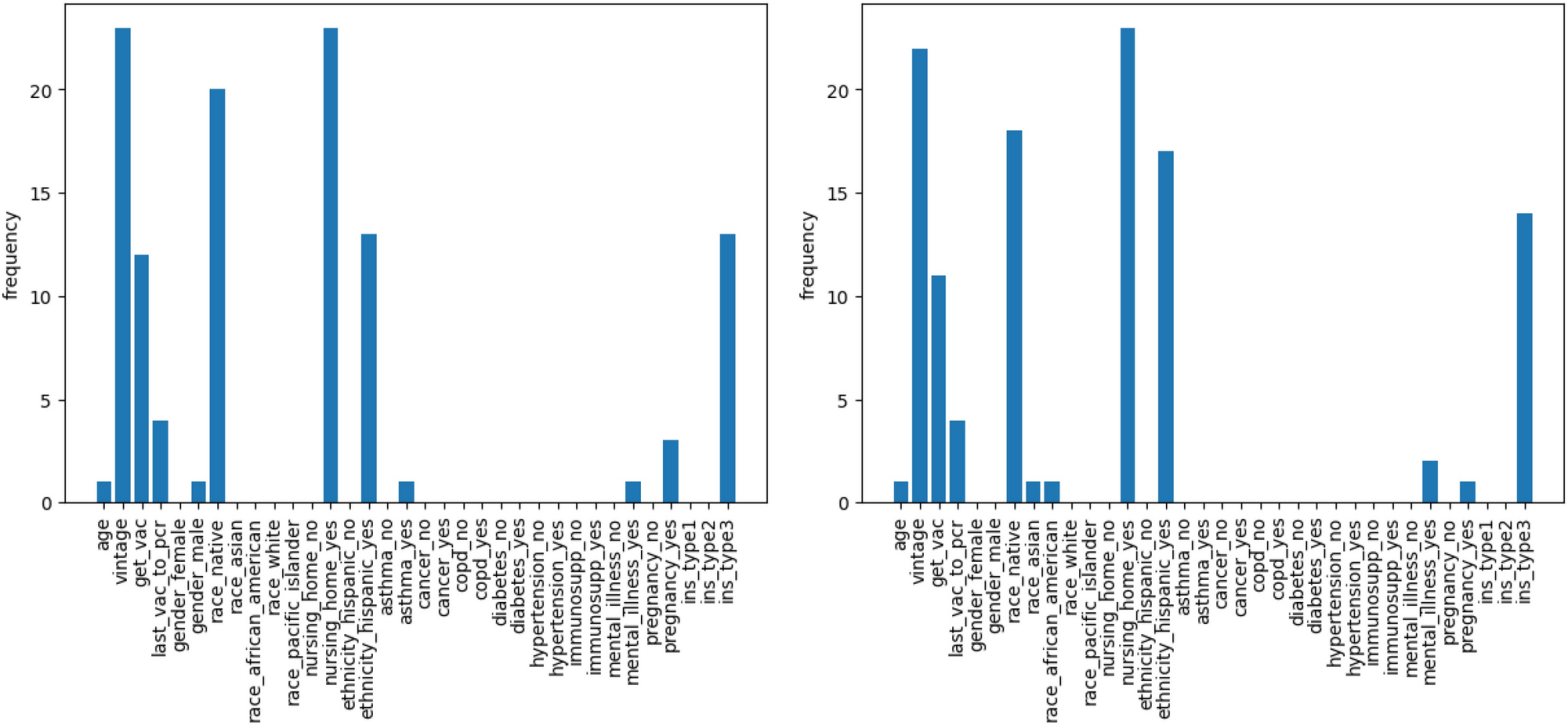
The number of months over the 25-month period for predictors are among the top five important static patient variables in LSTM(left) and CNN(right).

We apply the gradient-based method to evaluate the importance of features in the proposed RNN and CNN. The input data is fed through three distinct input channels: (1) Treatment and county infection variables, (2) Lab variables, and (3) Static variables. For each month, we calculate the gradients for the three channels and select the top five features with the highest averaged absolute gradients for each channel. We then summarize the counts of features appearing in the top five important features over 23 months in Figs. 3, 4, and 5 for our LSTM and CNN models.

Overall, both models produce comparable results in feature importance assessments. Specifically, in Fig. 3, both LSTM and CNN identify that local COVID-related features, such as population size, daily COVID-19 infection counts, and daily COVID-19 deaths, are the most relevant predictors. Additionally, individual weight is recognized as an important feature; for instance, both models highlight target weight as a key indicator for COVID-19 infection, with the CNN also identifying post-treatment weight as relevant.

In Fig. 4, both models agree that ferritin level, albumin, white blood cell (WBC) count, neutrophil-to-lymphocyte ratio (NLR), and hepatitis B surface antibody (anti-HBs) are the most important factors among lab variables. Additionally, the CNN model considers a wider range of features, including Transferrin saturation (TSAT) and lymphocytes.

For static features in Fig. 5, both LSTM and CNN detect that dialysis vintage, whether a patient is in a nursing home, whether a patient is Hispanic or Latino, and vaccine-related information such as whether a patient has been fully vaccinated and the number of days since the last vaccination are important features for COVID detection.

In summary, our study on feature importance highlights the most relevant indicators of SARS-CoV-2 infections. First, we confirmed that local COVID-19 conditions such as daily new cases and deaths play a critical role in assessing the risk of infections^17^. Second, we find that monitoring ferritin levels, white blood cell (WBC) counts, and oxygen levels is critical for dialysis patients during the COVID-19 pandemic not only due to their vulnerability and potential for severe disease progression but also for early prediction of SARS-CoV-2 infection. Elevated ferritin levels have been found consistently associated with severe COVID-19 outcomes, indicating inflammatory responses and the potential for cytokine storms, which are linked to higher mortality rates^21^. Additionally, increased WBC counts and altered neutrophil-to-lymphocyte ratio are important markers for disease severity and progression in COVID-19 patients, as these parameters reflect the body’s immune response to infection^22^. Monitoring oxygen saturation is also essential, as hypoxia can indicate respiratory insufficiency, a common and severe complication in COVID-19, necessitating immediate medical intervention to prevent mortality^23^. Therefore, these biomarkers are invaluable for early detection and management of complications in dialysis patients with COVID-19. Third, our study also confirms vaccination status, race, ethnicity, and nursing home are all effective COVID-19 predictors. Studies have shown that patients on maintenance dialysis have a significantly higher risk of COVID-19 related morbidity and mortality, making vaccination a top priority for this group^24^. Furthermore, the two subgroups, Hispanic and Native Americans have a higher contribution to the early prediction than other racial groups. Lastly, dialysis patients residing in nursing homes are particularly at risk due to the close quarters and frequent need for transportation to dialysis centers, which increases their exposure to the virus^25^. Effective infection control practices, including vaccination, regular screening, and coordinated care between nursing homes and dialysis centers, are essential to prevent outbreaks among this high-risk population^26^.

## Discussion

We have successfully developed two deep neural network models that leverage diverse data sources to proactively identify COVID-19 infections during the incubation period in maintenance hemodialysis patients. Through extensive monthly testing, we have demonstrated the clinical significance of our proposed deep learning models.

First and most notably, these models have shown substantial improvements compared to existing models. Overall, at a 20% false positive rate, the LSTM and CNN models could identify 66% and 64% of COVID-19 patients before their detection through RT-PCR COVID-19 tests. This is a significant improvement over the previous best-performing model (XGBoost), which identified 42%. Second, deep learning models require minimal feature engineering. In contrast to other machine learning models, such as XGBoost, which rely on complex feature engineering, both CNN and LSTM models treat individual variables as time series and extract time dependency signals through sophisticated architecture design. Consequently, LSTM and CNN models can uncover trends and patterns within clinical data that might not be evident through traditional feature engineering. Third, our study on feature importance identifies the most relevant predictors of SARS-CoV-2 infections, providing insights into effective infection control practices. We confirm previous findings that local COVID-19 conditions, vaccination status, race, ethnicity, and nursing home residency are all effective COVID-19 predictors. Additionally, our study reveals that monitoring ferritin levels, white blood cell (WBC) counts, and oxygen levels is also critical for the early prediction of SARS-CoV-2 infection.

Our study is subject to several limitations. Firstly, labeling positive COVID-19 diagnoses is based solely on the RT-PCR test date for confirmed positive patients. This approach might not fully capture the continuous and evolving nature of the COVID-19 infection. There could be instances before or after the RT-PCR test date that should also be labeled as positive, but the lack of comprehensive information makes it challenging to determine those periods accurately. Additionally, the variability in the timing of RT-PCR tests relative to the actual infection onset, influenced by factors like symptom presentation, introduces further uncertainty in the labeling process. Our choice of setting the prediction date three days before the index date is pragmatic, influenced by data availability and anticipated integration into clinical systems. Real-time data aggregation would be ideal, allowing the prediction date to align with the index date. This adjustment could enhance detection performance by incorporating the most recent and relevant lab and treatment data.

While our deep neural network models show promise, they have certain limitations. CNNs, for example, do not inherently consider temporal patterns, potentially leading to omitting important hidden patterns in time series data. On the other hand, LSTMs suffer from slow computation since their computation cannot be parallelized. Therefore, advanced techniques such as transformer models could be considered. In conclusion, while our study sheds light on early COVID-19 detection using deep learning methods, addressing these limitations and exploring advanced approaches could lead to even more accurate and impactful results in the field of clinical practice.

## Methods

### Multimodal data collection

Fresenius Kidney Care (FKC) is a dialysis organization encompassing approximately 2,400 dialysis clinics across all but one U.S. state. This substantial network offers dialysis treatments to around one-third of U.S. dialysis patients. FKC collects and maintains clinical, treatment, and laboratory data electronically. Our study focused on FKC patients with treatment records from November 2019 to February 2022. Patients suspected of harboring a SARS-CoV-2 infection at the outpatient dialysis clinics underwent RT-PCR testing to ascertain COVID-19 diagnoses.

Our investigation delved into a comprehensive array of demographic and socioeconomic factors for each patient. These factors include age, dialysis vintage, race, gender, education, employment status, and comorbidities such as hypertension, diabetes, congestive heart failure, and chronic obstructive pulmonary disease. Furthermore, we documented vaccination details, encompassing the vaccine type (Pfizer, Moderna, or Janssen) and the administration date for each patient.

Clinical data were meticulously recorded, spanning pre- and post-dialysis body temperature, pre- and post-dialysis systolic blood pressure, and interdialytic weight gain (IDWG). Additionally, treatment specifics such as treatment time, ultrafiltration volume, ultrafiltration rate, and Kt/V were extracted from electronic health records. Intradialytic data, comprising intradialytic blood pressure, heart rate, and ultrafiltration rate, were also meticulously documented.

Laboratory variables encompassed measurements taken roughly once a month, such as creatinine, BUN, albumin levels, neutrophil-to-lymphocyte ratio, and ferritin levels. Additionally, hemoglobin was monitored weekly. The comprehensive nature of these recorded variables enables a comprehensive exploration of the intricacies of dialysis patient health within our study framework.

Utilizing the home zip code of each patient, we extracted county-level infection data from the New York Times COVID-19 tracker (NYT COVID-19 tracker). This data encompassed daily records of new COVID-19 cases and COVID-19-related deaths^27^. For a comprehensive understanding of each county, we also extracted additional county information, including total population, population density, and the percentage of the population living in poverty, from the Census Bureau^28^. Furthermore, we introduced a new county-level feature termed ”Percentage of Contracting (PoC) COVID-19.” This feature was calculated for each U.S. county using a COVID transmission model^29^, which effectively gauges the daily risk a susceptible individual in that county faces to contract COVID-19. By incorporating this feature, we added a layer of granularity to our assessment of COVID-19 risk.

This research was executed according to a protocol evaluated by The Western Institutional Review Board (WIRB) under protocol number 20212859. WIRB concluded that the analysis of deidentified patient data fell under an exempt category, thus obviating the necessity for informed consent. The study was conducted in full compliance with the principles of the Declaration of Helsinki, ensuring the ethical conduct of the study.

### Confirmed cases and controls

We identified 41,515 dialysis patients who tested positive for COVID-19 between January 21, 2020, and February 28, 2022. These individuals were confirmed to have contracted the virus through at least one RT-PCR COVID-19 test during the study period. Only the first confirmed positive test date was considered for this study.

For the control group, we sampled a cohort of 114,119 patients who were negative for COVID-19. These patients were randomly selected from the entire pool of active FKC patients. The random sampling avoided potential selection bias. The criteria for inclusion in the negative cohort involved either having a negative RT-PCR test or not having been tested at all for COVID-19 during the observation period. Given there were more unaffected patients, we used a one-to-many (approximately one-to-three) matching to effectively utilize the data. Specifically, the matching will maximize the number of unaffected patients that can be used. Since we focus on a monthly updating strategy to emulate implementation in dialysis clinics, it is crucial to satisfy two constraints. One is to have sufficient positive and negative cases for each month since larger datasets generally enhance the prediction performance of all the machine learning methods, and the other is to maintain an approximately constant proportion of positive to negative cases over time so that testing results are comparable across different months. This matching procedure maximized the number of negative patients utilized, and at the same time maintain the ratio of positive and negative cases.

For COVID-19-positive patients, we define their ”index dates” as the dates they first received a positive RT-PCR test result. For patients who have never reported a positive RT-PCR outcome, their index dates are randomly selected from the collection of all index dates associated with positive patients.

**Fig. 6:**
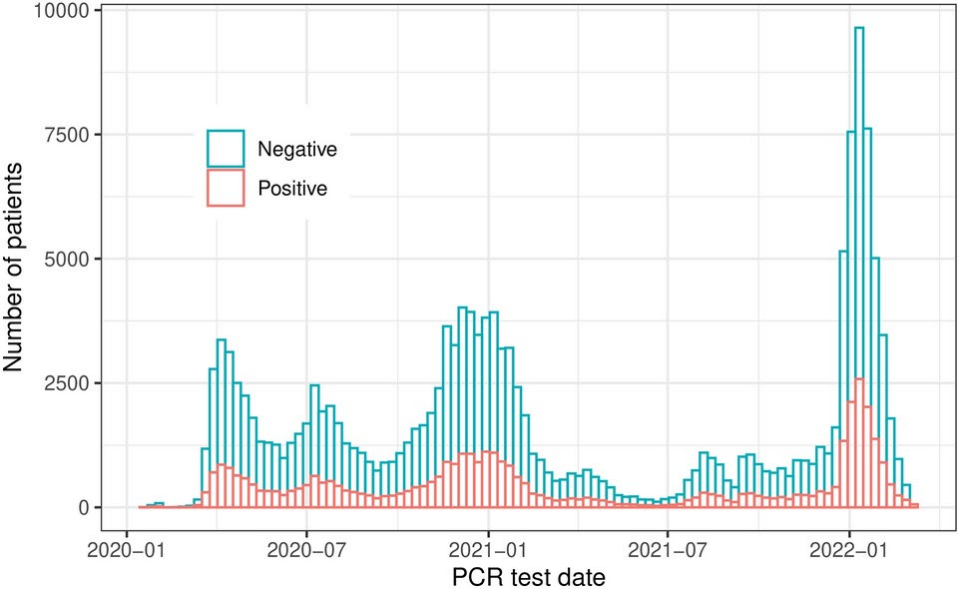
Patients distributions across the whole study period.

We applied specific inclusion criteria based on two key factors. First, patients need to have undergone a hemoglobin lab measurement within the time windows of 1-14 days and 31-60 days before their individual prediction date, which is set as 3 days before their index date. Second, we include patients who had received at least one dialysis treatment within 1-7 days and 31-60 days preceding their prediction date. These criteria were established to guarantee the inclusion of patients actively engaged in the FKC in-center dialysis program. Since hemoglobin measurements are conducted weekly for such patients, these criteria ensure the selection of patients with consistent and recent medical data. Figure 6 shows the patient distribution across the study period. We note the trend of COVID-19 cases is similar to that of the general population.

### Data processing

**Fig. 7:**
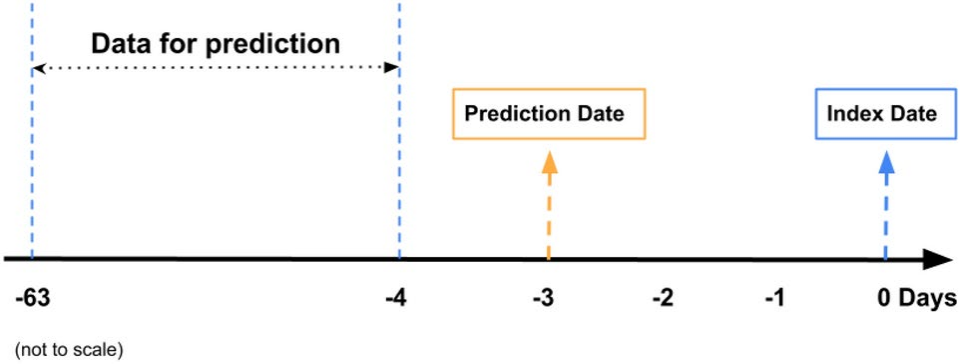
Data extraction and prediction timeline.

Our timeline setup follows the structure outlined in Monaghan et al.^15^ Specifically, we restrict our dataset to encompass data up to 4 days before the index date, corresponding to the anticipated RT-PCR test date (see Fig. 7). To ensure data quality, we eliminated outliers from laboratory and treatment measurements, which often arise from manual input errors. Examples include implausible values such as body temperatures below 70 $^{\circ }F$ or exceeding 120 $^{\circ }F$. For deep learning models, our data aggregation methods varied according to the nature of the variables. We employed daily measurements from each visit for treatment and county infection variables throughout the prediction period. Lab measurements were treated differently due to their less frequent nature, with the data time series aggregated to weekly averages. Meanwhile, for static data such as gender, race, and ethnicity, we generated 18 static features for each patient spanning the entire study period. Recognizing the decay of immunization effects post-vaccination, we incorporated the duration between the prediction date and the most recent vaccination date to capture this phenomenon. Lastly, we calculated the infection or death rate at the county level, expressed as the number of infections or deaths per million individuals. This metric serves to reflect the local epidemic dynamics. The categorical variables are one hot encoded. Feature engineering procedures for XGBoost, Logistic Regression, SVM and AdaBoost follows Duan et al.^17^ for comparison purpose. Namely, a time series variable is converted to three features by taking the average over two different periods, the normal period and the incubation period, and also forming the difference between the mean values in those two periods. The normal period is 31-60 days before the prediction date for every variable. For treatment and county infection variables, the incubation period is set to 1-7 days before the prediction date. For lab measurements, the incubation period is set to 1-14 days before the prediction date due to its less frequent schedule.

Another critical procedure we incorporated during the data processing phase deals with irregular sequence data. Due to the varying visit times for each patient, the need arises to transform sequence data into contiguous batches. This transformation is essential to align all sequences within a batch to a consistent predefined length. Consequently, sequences must be padded or truncated to conform to this standard length. Indeed, when dealing with irregular sequence data, it is essential to communicate to sequence-processing layers which timesteps in an input contain missing or padded values and, therefore, should be omitted during data processing. Both missing and padded values are replaced with 0, indicating the absence of information in those instances. The concept of masking is employed to ensure that our model can recognize and account for the presence of missing or padded values. Masking involves introducing a placeholder value subset within the input sequence. This signals the system to identify and disregard these padded values during the data processing phase. By incorporating masking, the system can accurately process sequences with varying lengths while appropriately accounting for and excluding padded values.

### Training and testing

Based on our preliminary analysis, we meticulously curated a selection of distinct features for our predictive analysis. These encompass 28 treatment-related and local county variables, 25 laboratory features, and 18 demographic attributes. All these features were considered up to 4 days before the index date. An exhaustive breakdown of these features can be found in Tables 1, 2, and 3.

**Fig. 8:**
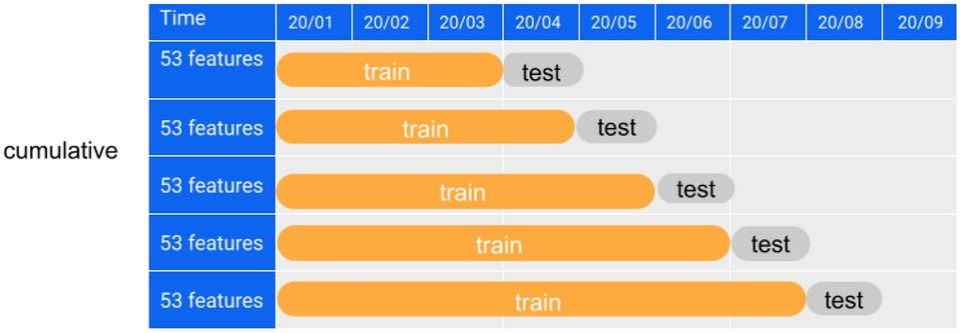
Cumulative training strategy.

We selected these features up to 4 days before the index date. The index date corresponds to the date when patients receive their test results. In contrast, the prediction date is when we leverage these features to forecast the likelihood of identifying a SARS-CoV-2 infection within the following three days or beyond. We have employed a monthly updating strategy, mimicking the implementation process typically followed in dialysis clinics. For example, when making predictions for August 2020, we exclusively employed data from before August 1, 2020, as the training dataset. As a result, the evaluation of performance for August 2020 remains entirely outside of the training data, closely mimicking real-world conditions. In our study, we compared two distinct training strategies outlined in Fig. 8 and Fig. S1 in the Supplement. The first approach uses all data from January 2020 to the testing phase, while the second uses 3-month data before the testing phase. Since all DNN models based on cumulative data perform better than those based on 3-month data, we only include results based on cumulative data in the main text and present results based on 3-month data in the Supplementary Material. We adopted binary cross-entropy as the loss function throughout the training, steering the learning process toward enhanced predictive capability.

### Software and machine configuration

Python was used for model training with CNN and RNN, while R was used for data cleaning and for training Logistic Regression, SVM, AdaBoost and XGBoost models. The machine used was a Windows 10 system with a Dual 2.5 GHz Intel Xeon E312xx (Sandy Bridge) CPU and 128 GB of memory. Prediction for a single observation from a patient takes less than 5 seconds for all models on our machine, based on speed tests conducted on over 5,000 patients.

### Evaluation metric

We employ two key metrics to assess performance over the period spanning April 2020 to February 2022: the monthly out-of-sample Area Under the Receiver Operating Characteristic Curve (AUC). Furthermore, we compute the overall metrics of the Area Under the Curve (AUC) and the Precision-Recall Curve (PRC). These aggregated metrics comprehensively assess a model’s performance throughout the study.

### Recurrent neural network (RNN) and long short-term memory (LSTM)

The input to an RNN is a multivariate time series. Following notations in Lea et al.^30^, let $X_t\in R^{F_0}$ be the input feature vector of length $F_0$ for time step t where 0 < t $\le$ T. In classification setting, if the classification task is to get the last output for the *i*-th patient $y_{i}$ only (known as many-to-one RNN) and the label for each sequence is given by $y_i \in$ {1, . . . , C}, where C is the number of classes, then the goal of an RNN is to predict the class label $\hat{y}_{i}$ and minimize the cross entropy between true labels and the distribution of probabilities of predicted classes. The average loss when classifying the whole training set of *D* is

$$\begin{aligned} \mathscr {J}(W)= -\frac{1}{N}\sum _{i=1}^{N}\sum _{c=1}^{C} {y_{i}}^{(c)} \cdot \log {\hat{y}_{i}^{(c)}} , \end{aligned}$$

where *N* is the total number of objects in *D*, ${\hat{y}_{i}}^{(c)}$ is the predicted probability of $y_{i}$ belonging to class *c*, and ${y_{i}}^{(c)}$ equals to 1 if it belongs to class *c* and 0 otherwise, and *W* denotes the set of weights to be learned by the network.

RNN aims to extract temporal behavior between elements in an input sequence and produce outputs dependent on the sequence’s previous information. At each time step t, an RNN takes in an input $\textbf{x}_t$, updates a hidden state $\textbf{h}_t$ and produces an output $\textbf{y}_t$ as follows^31^:

$$\begin{aligned} \mathrm {\textbf{h}_t = tanh(\mathbf {W_h[h}_{t-1},\mathbf {{x}}_t])+\mathbf {b_h})} , \; \; \mathrm {\textbf{y}_t = softmax(\mathbf {W_yh}_{t}+\mathbf {b_y})} , \end{aligned}$$

where tanh and softmax are activation functions, $\mathbf {b_h}$ and $\mathbf {b_y}$ are biases, and $\mathbf {W_h}$ and $\mathbf {W_y}$ are the recurrent weight matrix for the hidden state $h_t$ and the recurrent weight matrix for the output $y_t$.

**Fig. 9:**
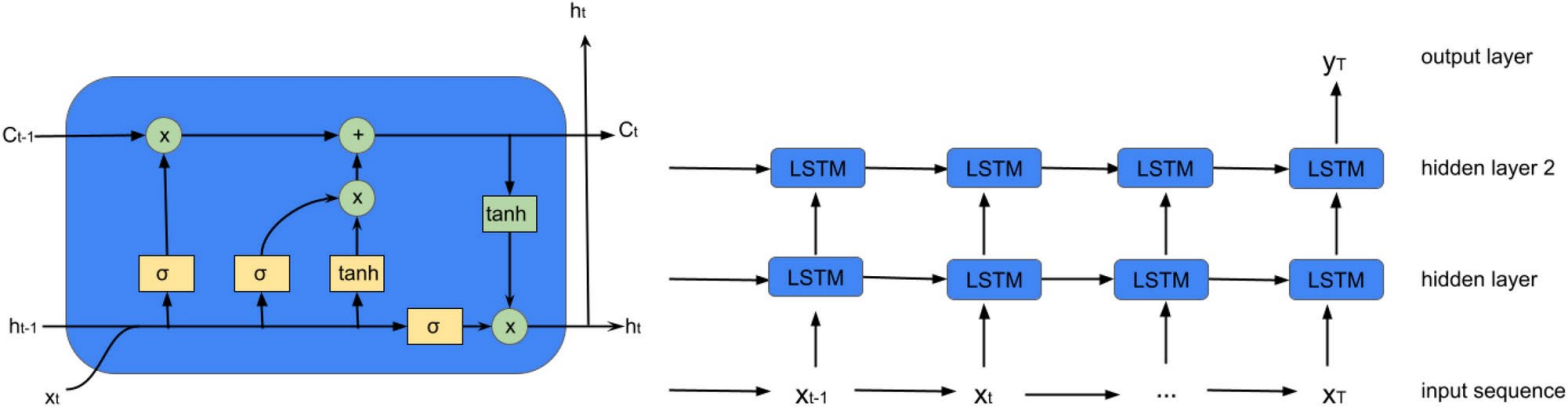
LSTM inner structure (left) and many to one LSTM model structure (right).

LSTM is an RNN that can deal with longer sequences without a vanishing gradient problem. First introduced by Hochreiter and Schmidhuber^2^, LSTMs solve the vanishing gradient problem by including different gates in RNNs. The left panel of Fig. 9 shows the workflow at each timestep t. The yellow boxes are ”gate layers” and the green boxes represent the operational rules applied, including operations such as addition, multiplication, and applying a hyperbolic tangent (tanh) function.

At each time step t, LSTMs employ three gates: the forget gate, the input gate, and the output gate, to filter information from the combination of the hidden state $\textbf{h}_{t-1}$, cell state $\textbf{C}_{t-1}$, and input element $\textbf{x}_{t}$, as follows:

1 $$\begin{aligned} {{\textbf{f}}_t = \sigma (\textbf{W}_{f}\cdot [\textbf{h}_{t-1}, \textbf{x}_{t}]+\textbf{b}_f)} , \; \; {{\textbf{i}}_t = \sigma (\textbf{W}_{i}\cdot [\textbf{h}_{t-1}, \textbf{x}_{t}]+\textbf{b}_i)} , \; \; {\textbf{o}_t = \sigma (\textbf{W}_{o}\cdot [\textbf{h}_{t-1}, \textbf{x}_{t}]+\textbf{b}_o)} , \end{aligned}$$

where $\sigma$ is a sigmoid function, and the hidden state and cell state are updated as follows:

2 $$\begin{aligned} {{\tilde{\textbf{C}}}_t = tanh(\textbf{W}_{C}\cdot [\textbf{h}_{t-1}, \textbf{x}_{t}]+\textbf{b}_C)} , \; \; {{\textbf{C}}_t = \textbf{f}_t \cdot \textbf{C}_{t-1} + \textbf{i}_t \cdot {\tilde{\textbf{C}}}_t} , \; \; {{\textbf{h}}_t = \textbf{o}_t \cdot tanh(\textbf{C}_t)} . \end{aligned}$$

### One dimensional convolutional neural network (1D-CNN)

CNN usually consists of several major components: (1) a convolutional layer (CONV) to perform convolution operations on the time series of the preceding layer with various convolution filters. The output is called a feature map or activation map; (2) a pooling layer (POOL) for a downsampling operation, typically applied after a convolution layer. POOL layers reduce the information in each feature provided by CONV layers but maintain the most important information. The most common choices are max and average pooling, indicating the calculation of maximum and average values over the values inside the POOL, respectively; and (3) a fully connected layer (FC) takes a flattened feature as input and outputs the distribution of class probabilities in classification settings. Usually, raw time series input will go through the combinations of CONV and POOL layers several times before being fed into FC layers for final prediction. Non-linear functions, e.g., sigmoid, ReLU, and tanh, are added in CONV and FC layers to introduce non-linearity into the network. Details can be found in Zhao et al.^4^

For each of the *L* convolutional layers in the encoder, a set of 1D filters that capture how the input signals evolve over the course of an action are applied^30^. The filters for each layer are parameterized by tensor $W^{(l)} \in \mathbb {R}^{F_l \times d \times F_{l-1}}$ and biases $b^{(l)} \in \mathbb {R}^{F_l}$, where *l*$\in$ {1,...,L} is the layer index and *d* is the filter duration. The *i*-th element of the activation ${\hat{\textbf{E}}}^{(l)}_{t} \in \mathbb {R}^{F_l}$ of the *l*-th layer is a function of the activation matrix $\textbf{E}^{(l-1)} \in \mathbb {R}^{F_{l-1}\times T_{l-1}}$ of the previous layer as follows^32^:

3 $$\begin{aligned} {{\hat{\textbf{E}}}^{(l)}_{i,t}} = f\mathrm {\Biggl (b^{(l)}_{i}+\sum _{t'=1}^{d} {\biggl \langle W^{(l)}_{i,t',.}, E^{(l-1)}_{.,t+d-t'} \biggr \rangle }\Biggr )} \end{aligned}$$

for each time *t*, where *f* is the activation function.

CNNs are powerful deep learning methods for time series analysis due to the advantage of parallel computation^33^. A simple convolutional architecture outperforms canonical recurrent networks such as LSTMs across various tasks and datasets while demonstrating longer effective memory^34^. However, CNNs do not consider temporal patterns and may result in missing significant patterns hidden in time series data.

### Model architecture

**Fig. 10:**
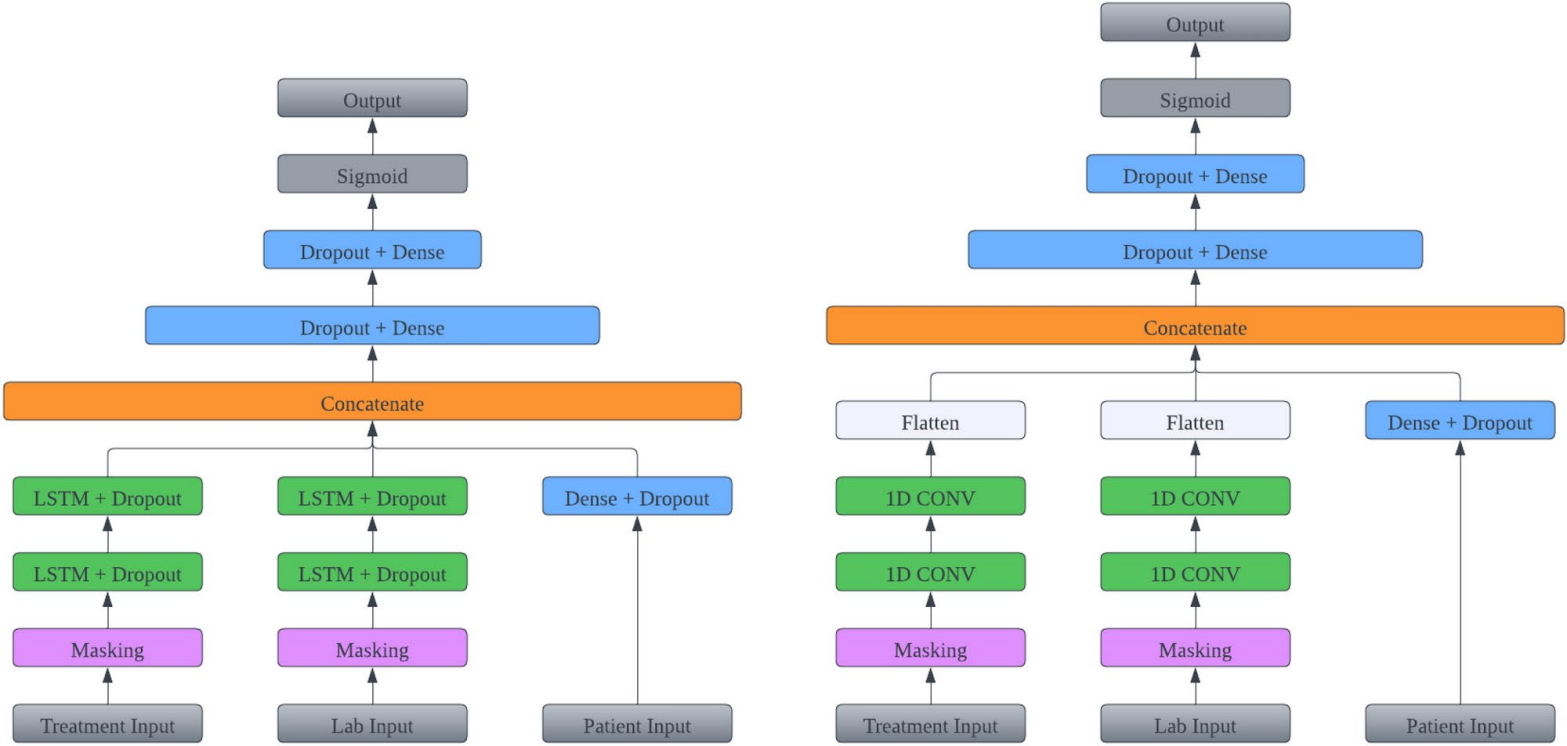
Multi-inputs LSTM models architecture (left) and Multi-inputs CNN models architecture (right).

We introduce two deep-learning architectures in this section for classifying COVID-19-positive patients. These models encompass an LSTM-based and a CNN-based architecture in Fig. 10. Because patients undergo dialysis treatment several times a week and have lab test roughly once a month. The collection frequency of treatment variables and lab variables are drastically different. This inhomogeneity in different longitudinal measurements pose a great challenge in applying deep learning methods. A simple masking or padding for the low frequency data is unlikely to work well. Unlike typical RNN and CNN models, we designed an architecture with multiple parallel neural blocks that is tailored to efficiently process static data and time series data with different frequencies separately. We now provide details for each layer and its respective function within both models.

*Input layers*: In Fig. 10, two distinct multivariate time series data sets, Treatment and Lab, are employed as separate inputs necessitated by different collection frequencies in these data sets. The Treatment input comprises 28 variables, encompassing 23 treatment-related measurements and 5 local county variables. Summary statistics of these Treatment variables are provided in Table 2. Conversely, the Lab input channel consists of 25 variables with their summary statistics presented in Table 3. The Patient input remains consistent across time, serving as a static channel. This channel provides information on race, ethnicity, sex, vintage length, age, and the presence of specific diseases. By introducing dummy variables for non-numeric attributes, 34 static variables were generated for the Patient input. These variables are summarized in Table 1. These three input channels, namely Treatment input, Lab input, and Patient input, collectively constitute our input data. They are depicted as the gray boxes at the bottom of Fig. 10.

*Padding and masking*: Upon obtaining the three input data channels, the Treatment and Lab channels undergo a crucial pre-processing step: they are directed through a masking layer before proceeding into the LSTM or CNN layers. This step is vital due to the variability in patient clinic visit times, leading to diverse time series sample lengths. Batch learning requires uniform input sequence lengths. To achieve this uniformity, a technique known as post-padding is introduced. This technique entails appending extra placeholder values to the end of sequences, ensuring consistent length across all samples. Then, we apply a masking layer to the sequence-processing LSTM or CNN layers. This masking layer intelligently ignores specific timesteps designated as placeholders during the data processing phase. As a result, these padded segments do not contribute to the learning process, preserving the fidelity of the data and allowing for effective batch learning.

*LSTM layers + dropout*: In the RNN block, two stacked LSTM blocks, each with a filter size of 64, are employed. Choosing two LSTM layers within each block increases model complexity, improving performance during the evaluation phase. Each block includes an LSTM layer with a dropout rate of 0.2, a recurrent dropout rate of 0.2, and a tanh activation function, followed by a sigmoid recurrent activation function, as described by (1) and (2). A dropout rate is a fraction of the units to drop for the linear transformation of the inputs, which means that a certain fraction of input $x_t$ is dropped, and the calculation from $x_t$ to $h_t$ is also dropped. A recurrent dropout rate is a fraction of the units to drop for the linear transformation of the recurrent state, which means that a certain fraction of input $h_{t-1}$ is dropped and thus calculation from $h_{t-1}$ to $h_t$ is also dropped. A typical RNN model structure is presented in Fig. 9, where $x_t$ is the input data and $h_t$ is the hidden layer at time t. The regular dropout is applied on vertical arrows whereas the recurrent dropout is applied on horizontal arrows respectively in Fig. 9.

*CNN layers + global pooling layer*: The CNN-based classification model outlined on the right of Fig. 10 shares most components with the RNN architecture. A fundamental distinction in the CNN-based model lies in the utilization of fully convolutional networks, specifically employing temporal convolutions as primary feature extractors. The fully convolutional block in the CNN model consists of two consecutive temporal convolutional blocks, each characterized by filter size 64. Each block comprises a temporal convolutional layer, immediately succeeded by a Rectified Linear Unit (ReLU) activation function. Notably, the architecture leverages fully convolutional networks to effectively extract relevant features from the input data. A notable deviation in this model is the incorporation of global average pooling within the Flatten layer. This step is implemented to reduce dimensionality following the final convolutional block. Global average pooling computes the average across all time steps within a time series, effectively flattening the data shape from (batch size, time steps, features) to (batch size, features). This process aids in condensing the extracted information while preserving crucial features for subsequent classification.

*Dense + dropout layer*: In parallel, non-temporal data (Patient) is passed through a dense layer. For each dense layer, it implements the operation, $\mathrm {\textbf{y} = f(\textbf{w}\cdot \textbf{x}+\textbf{b})}$, where $\textbf{x}$ is the input data, $\textbf{y}$ is the output from this dense layer, $\textbf{w}$ are the weights, $\textbf{b}$ is the bias learned in the layer, and *f* is the activation function.

*Concatenate layer*: The output generated by the dense layer from the ”Patient” input is combined with the output from either the LSTM or CNN blocks. This combination is achieved using a concatenate layer, which operates on inputs having the same shape except for the concatenation axis. The outcome of this process is a single tensor formed by concatenating all input tensors. For instance, following the flatten layer, the Treatment CNN and Lab CNN outputs possess dimensions of (batch size, treatment features) and (batch size, lab features) respectively. Simultaneously, the Patient dense layer generates an output with dimensions of (batch size, patient features). Upon concatenation, these individual outputs merge to form an output with dimensions of (batch size, treatment features + lab features + patient features). This strategic concatenation facilitates the incorporation of vital insights from each input channel while upholding their distinctive attributes.

*Dense and classification layers*: The concatenated output will undergo a series of dense layers before being directed to a sigmoid classification layer.

## Supplementary Information

Supplementary Information.

## Data Availability

The complete dataset discussed in this article is not available for public access due to the inclusion of protected health information. Partial data are available at the RADx Data Hub https://radx-hub.nih.gov.
