# Supplementary material for: Predicting SARS-CoV-2 infection among hemodialysis patients using deep neural network methods
Source: Sci Rep. 2024 Oct 9;14:23588. doi: 10.1038/s41598-024-74967-4 (PMC11464512; doi:10.1038/s41598-024-74967-4)
Supplement: Supplementary file 1 — Supplementary Information. [file 41598_2024_74967_MOESM1_ESM.pdf]

Predicting SARS-CoV-2 Infection Among Hemodialysis Patients Using Deep Neural Network Methods

Supplementary Material

Models Based on 3-Month Data

In the supplement, we present an alternative training strategy that uses 3-month data. Compared to the cumulative training strategy that uses all data from the beginning of 2020 presented in the main text, 3-month strategy uses data within three months before the testing period, as shown in Figure S1. For example, for the prediction in August 2020, we used data from the previous three months (from May 1 to July 31, 2020) as the training sample.

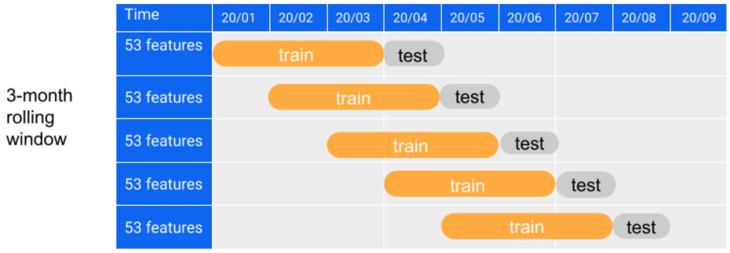

Figure S1. 3-month training strategy.

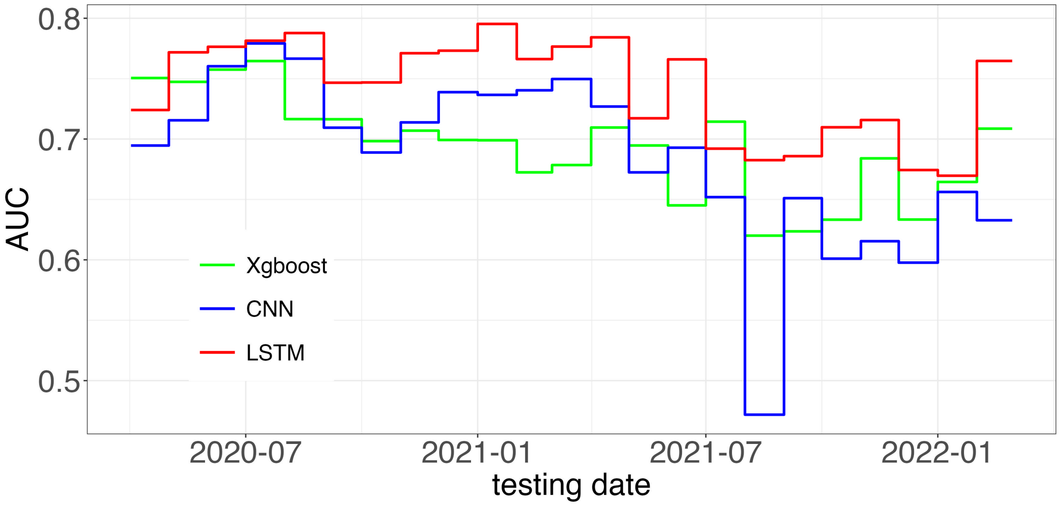

Figure S2. Monthly AUCs from LSTM, CNN, and XGBoost models based on 3-month data.

The LSTM model stands out as the most effective among the three. The AUC values for this model demonstrated a consistent stability of around 0.75 until April 2021. There was a decline to approximately 0.7 between May 2021 and January 2022 before a subsequent increase back to 0.75 in February 2022. The CNN model showed comparability to the baseline XGBoost model, except for a noticeable performance dip in August 2021, where the AUC value plummeted below 0.5.

When comparing different training strategies, it is evident that the models based on cumulative data outperform those based on 3-month data, especially from April 2021 to January 2022. As seen in the distribution of positive patients in Figure 3, the number of positive cases was relatively low during this period. However, deep neural network models like CNN and LSTM perform better when trained on larger datasets. The cumulative learning strategy leverages patient information up to the current training point, ensuring a larger data pool to enhance the training process. This translates to improved performance of both LSTM and CNN models, particularly during periods of lower positive case numbers. During the final month of the study, characterized by a substantial increase in positive cases due to the Omicron variant, the advantage of models based on cumulative data becomes less pronounced.

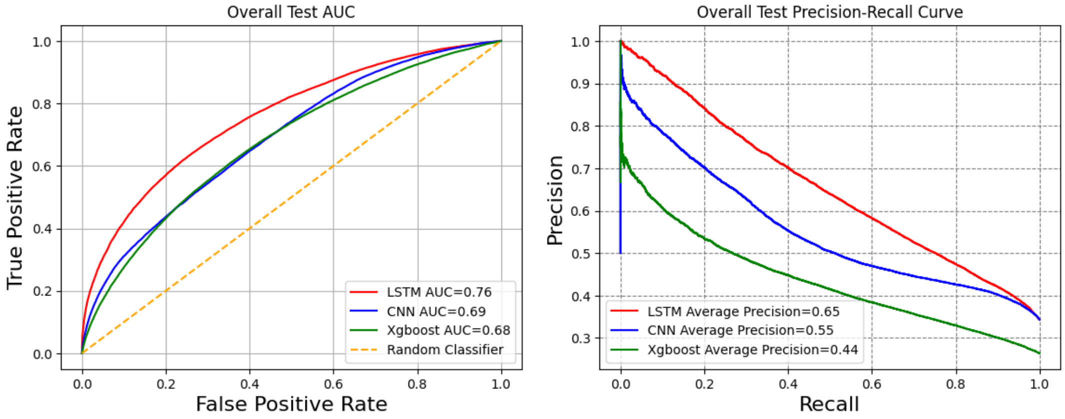

Figure S3. Overall testing performance is calculated with aggregated monthly predictions. We compare receiver operating characteristic curves (AUCs) (on the left) and precision-recall curves (PRCs) (on the right) for LSTM, CNN, and XGBoost models based on 3-month data.
